# Real-Time AI-Based ACR TI-RADS Classification of Thyroid Nodules on the Ultrasound Console: A Prospective Agreement Study in a Region with Historical Iodine Deficiency

**DOI:** 10.3390/diagnostics16203265

**Published:** 2026-10-09

**Authors:** Marcel Wehmann, Martin Freesmeyer, Christian Kühnel, Falk Gühne, Philipp Seifert

**Affiliations:** Clinic of Nuclear Medicine of the Jena University Hospital, Am Klinikum 1, 07747 Jena, Germany; marcel.wehmann@med.uni-jena.de (M.W.); philipp.seifert@med.uni-jena.de (P.S.)

**Keywords:** thyroid nodules, ACR TI-RADS, ultrasound, artificial intelligence, risk stratification, observer agreement

## Abstract

**Background/Objectives**: To assess the agreement between an artificial intelligence (AI)-based decision support system (DS System), applied in real time directly on the ultrasound console, and an expert panel for ACR TI-RADS classification of thyroid nodules (TNs) in a region with a history of iodine deficiency and a consequently high prevalence of TNs. **Methods**: A single-center prospective study including 118 consecutive patients (with 276 TNs) referred for thyroid ultrasound between March and May 2024. DS System ultrasound features, ACR TI-RADS classifications (TR1–TR5) and management recommendations were recorded per TN, with and without the ‘AI-Adapter’ (AI-based modification of the TI-RADS score), and compared to the consensus assessment of a TI-RADS expert panel (paired tests of marginal homogeneity, Cohen’s unweighted and weighted κ; all analyses adjusted for clustering of TNs within patients). **Results**: No malignancy was identified during 24-month follow-up (composite reference standard; cytology or histology in six TNs). The DS System could be applied to all TNs without technical failure. Marginal distributions differed between the expert panel and the DS System for all ultrasound feature categories except echogenicity (*p* = 0.23). Interrater agreement ranged from poor (“lobulated or irregular”, κ = −0.08) to substantial (“cystic or almost completely cystic”, κ = 0.62), most frequently slight to fair. TI-RADS classifications showed slight agreement with (κ = 0.04; linear-weighted κ = 0.14) and without AI-Adapter (κ = 0.13; linear-weighted κ = 0.21). The AI-Adapter changed the ACR TI-RADS score by −1.01 ± 1.04 points. Management recommendations showed fair agreement with (κ = 0.23) and without the AI-Adapter (κ = 0.39). Although the number of TR5 ratings was similar (expert panel: 27; DS System: 26 without and 30 with the AI-Adapter), only seven and eight of the 27 TR5 TNs of the expert panel, respectively, were also rated TR5 by the DS System. **Conclusions:** Real-time application of the DS System on the ultrasound console was technically feasible, but agreement with an expert panel was low. As all TNs were benign, diagnostic accuracy and clinical safety could not be assessed. Validation in cohorts including malignant TNs is required before the DS System can be considered a substitute for expert assessment in regions with a high prevalence of TNs.

## 1. Introduction

The prevalence of thyroid nodules (TNs) exhibits significant variation on a global scale, ranging from 2% up to ~50%, contingent on dietary iodine intake [1,2]. A substantial corpus of evidence exists that proves the historical iodine deficiency and its resultant high prevalence of TNs within the German population [3,4,5,6]. While the majority of these findings are benign, a significant diagnostic challenge lies in reliably identifying the rare malignant cases [7,8]. Precise differentiation is crucial in order to avoid unnecessary invasive procedures while enabling early treatment of malignancies [9,10]. To accomplish this objective, ultrasound is an indispensable instrument [11,12]. A variety of risk stratification systems (RSS) have been developed for the purpose of standardizing TNs diagnosis [13,14]. These systems are based on the sonographic assessment of morphological criteria and aim to improve the diagnostic accuracy. In many studies, American College of Radiology (ACR) Thyroid Imaging Reporting and Data System (TI-RADS) has been demonstrated to be stable and reliable [14,15,16].

However, when evaluating ultrasound images, significant interobserver variability exists, which can limit the reproducibility and comparability of findings [17,18]. Recently, artificial intelligence (AI)-based decision support (DS) systems have gained increasing attention in medical imaging, particularly for risk stratification tasks [19,20]. In thyroid diagnostics, numerous studies have demonstrated the potential of AI to analyze ultrasound images of TNs, yielding promising diagnostic performance [21]. These approaches have shown potential to improve risk assessment and support clinical decision-making [22]. The database of advanced systems used for research purposes, such as AIBx, ThyNet, AI-Thyroid, and ThyGPT, is constantly growing [23,24,25,26]. However, only a few systems have been implemented in routine clinical practice; these are listed in Table 1.

Koios DS Thyroid (Koios Medical, Inc., Chicago, IL, USA) offers the broadest range of integration options among the clinically implemented AI-based DS Systems for TI-RADS classification of TNs. In addition to its capacity for Picture Archiving and Communication System (PACS) integration on a workstation, the DS System can be utilized directly during live ultrasound on the GE LOGIQ E10 console (GE HealthCare, Chicago, IL, USA). The system requires a secure network connection to the manufac-turer’s server software (see Section 2.2). Conversely, S-Detect for Thyroid (Samsung Medison Co., Ltd., Seoul, Republic of Korea), as a hardware integration, is exclusively built into Samsung ultrasound devices and cannot be used in conjunction with PACS. Am-CAD-UT Detection (AmCad BioMed Corporation, Taipei, Taiwan) does not provide integration into ultrasound console environments. The PIUR tUS Infinity system (PIUR IMAGING GmbH, Vienna, Austria) is compatible with multiple ultrasound systems but requires additional secondary hardware (i.e., a PC) for image evaluation. Direct integration with ultrasound consoles or PACS workstations is not available [27,28,32,33,34].

According to the manufacturer, the data underlying the Koios DS Thyroid system is primarily derived from U.S. patient cohorts, where the prevalence of TNs is relatively low, especially in comparison to Germany [1,29]. Previous manufacturer-independent studies investigating the performance of this system were likewise carried out in regions with lower TN prevalence (Spain and the U.S.) [29,30,31,35,36]. The first AI-based DS data from a German cohort were recently published using the PIUR tUS Infinity system [34]. However, all the mentioned studies were carried out retrospectively. With regard to studies on the Koios DS Thyroid system, previous data were evaluated using a PACS workstation. The absence of prospective data on real-time use at the ultrasound console is a notable limitation in the literature.

The transferability of AI-based DS Systems to such populations cannot be taken for granted. Beyond the lower proportion of malignant TNs, which inevitably affects predictive values, the sonographic spectrum of TNs in formerly iodine-deficient regions differs from that of the cohorts in which most training data were acquired: multinodular goiters with multiple, partly adjacent or confluent TNs are common, and a considerable proportion of TNs are autonomously functioning [3,4,5,8]. Long-standing nodular goiter is, moreover, frequently accompanied by regressive changes such as cystic degeneration, fibrosis and calcifications, and the background parenchyma may be heterogeneous, for example in nodular goiter or coexisting autoimmune thyroiditis. Because echogenicity is defined relative to the adjacent thyroid parenchyma and nodule margins have to be delineated against it, such conditions may impair both the automated extraction of individual ultrasound features and the AI-based risk estimate (spectrum or domain shift). These considerations are hypothesis-generating; whether and to what extent they affect the performance of the DS System has not yet been investigated.

This study aimed to prospectively assess the agreement between the Koios DS Thyroid system (hereinafter referred to as the ‘DS System’), applied in real time directly at the ultrasound console, and a TI-RADS expert panel regarding ultrasound features, ACR TI-RADS classification and management recommendations in a region with a history of iodine deficiency and a consequently high prevalence of TNs. To our knowledge, this is the first prospective evaluation of the DS System in real-time use on the ultrasound console and the first evaluation in a formerly iodine-deficient population. The study was designed as an agreement study. Given the nature of the clinically led treatment pathways, which are not influenced by the research approach of our study, the assessment of diagnostic accuracy against a gold standard, such as cytological or histological results, was not an objective. Instead, clinical follow-up according to the local standard of care was used to characterize the nature of the TNs.

## 2. Materials and Methods

### 2.1. Study Design and Patients

This prospective, single-center, observational study included all consecutive patients in whom at least one TN was detected during a thyroid ultrasound examination at our institution between March and May of 2024 (the duration of the free trial for the investigated DS System). No additional inclusion or exclusion criteria were applied. In particular, TNs were not selected with respect to their sonographic appearance, functional status or presumed dignity. Informed consent was obtained from all subjects (or their legal guardians) involved in the study. The study was conducted in accordance with the Declaration of Helsinki. It was also approved by the Ethics Committee of Jena University Hospital in Germany (approval number 2024-3207-Daten, 12 January 2024).

The pseudonymized patient data collected included demographic and health-related information, including sex, age, medical history, thyroid status (organ size, number of TNs, thyroid medication, prior treatment modalities, and laboratory test results). In instances where fine-needle aspiration (FNA) was performed, the resulting outcomes were meticulously documented, with reference to the 2017 Bethesda system [37].

The nature of all TNs (benign versus malignant) was assessed during a 24-month follow-up period (until May of 2026). When no cytological or histological results were available, the TNs were considered to be benign if scintigraphy clearly demonstrated autonomously functioning thyroid nodules (AFTNs) or if sonographic stability was documented over the entire follow-up period [38,39,40]. Indications for FNA or surgery were determined within the clinical routine according to the local standard of care, independently of the study and of the ACR TI-RADS recommendations (see Section 4.5).

### 2.2. Data Assessment

The DS System under investigation (Koios DS Thyroid, software version 3.3.2; Koios Medical, Inc., Chicago, IL, USA) was installed on the ultrasound console (GE LOGIQ E10s, software version R3; GE HealthCare, Chicago, IL, USA) by the local GE HealthCare service team. The system was implemented for all detected TNs during the ultrasound examination, which was performed with a linear transducer (ML4-20VN; GE HealthCare, Chicago, IL, USA). All ultrasound parameters (e.g., frequency, depth, and gain) were individually optimized with respect to the circumstances of the respective patients. For each TN, the examining physician (M.W.) acquired a transverse and a longitudinal image of the lesion of interest and regularly placed the diameter measurements encompassing the entire TN on each image. The DS System then automatically assessed the ACR TI-RADS ultrasound features and returned the resulting TI-RADS classification without and with AI-Adapter. The automatically assigned features were not modified by the operator. The DS System results were then saved to the patient’s file. The AI-Adapter is a modification of the ACR TI-RADS score derived from an algorithm trained using machine learning techniques (observed range in this cohort: −2 to +2 points); its specific methodology remains undisclosed. At the timepoint of investigation the DS System required a secure network connection to the manufacturer’s server software (version 3.3.2, locally installed on a separate PC), via which no identifying patient data were transmitted. According to a non-public manufacturer’s training protocol, the algorithm was trained on more than 350,000 histologically verified TNs. The software version remained unchanged throughout the study period. Processing time, additional examination time and the number of repeated analyses were not systematically recorded.

In a separate session (9–12 months later), an expert panel of TI-RADS specialists assessed all included TNs on a PACS workstation (Centricity Universal Viewer, version 7.0 SP1.0.1; GE HealthCare, Chicago, IL, USA) using the same transverse and longitudinal images that had been analyzed by the DS System. The TNs were rated by consensus from the outset. No independent readings by the individual panel members were performed beforehand, so that inter-expert agreement could not be determined. The panel members were blinded to the DS System results as well as to scintigraphic, cytological, histological and follow-up findings). The panel included a DEGUM (German Society for Ultrasound in Medicine)-certified thyroid sonographer with 5 years of clinical experience (M.W.) and a TI-RADS expert with 15 years of experience in thyroid imaging and more than 20 TI-RADS related publications (P.S.). For both the expert panel and the DS System, management recommendations (no follow-up, follow-up, FNA) were derived from the TR category and the maximum TN diameter according to the ACR TI-RADS size thresholds [16].

### 2.3. Data Analysis and Statistics

Data were documented in Microsoft Excel (Version 2016, Microsoft Corporation, Redmond, WA, USA) and transferred to RStudio (Version 2026.07.1, Posit Software, PBC, Boston, MA, USA, https://posit.co/products/open-source/rstudio, last access: 25 July 2026) with R (version 4.4.3; R Foundation for Statistical Computing, Vienna, Austria) for statistical analyses.

Because each TN was rated by both the expert panel and the DS System, all comparisons were performed as paired analyses. Differences in the marginal distributions (i.e., in the frequencies of the individual categories) were tested with the McNemar test for binary subcategories and with the Stuart–Maxwell test of marginal homogeneity for multi-category features [41,42]. To account for the clustering of TNs within patients, both tests were computed with a cluster-robust variance estimator based on patient-level sums of the individual discordances. For binary subcategories, this corresponds to the clustered McNemar test described by Durkalski et al. [43]. The same approach was used to compare the DS System ratings with and without AI-Adapter. Because the subcategories of echogenic foci are not mutually exclusive, these were tested separately as binary variables only.

Agreement at the level of the individual TN was quantified with Cohen’s unweighted κ for each ultrasound feature and, after dichotomization (present versus absent), for each subcategory. For the ordinal ACR TI-RADS categories (TR1–TR5) and the ordinal management recommendations (no follow-up, follow-up, FNA), linear-weighted κ was additionally calculated, which gives partial credit to disagreements between adjacent categories [44]; quadratic-weighted κ was calculated as a sensitivity analysis. Unweighted κ was retained as the primary measure because clinical management depends on the exact TR category and because the ACR TI-RADS categories are not equidistant (e.g., TR4 spans three point values, 4–6 points), so that distance-based weights cannot be assumed to reflect equal clinical consequences of adjacent-category disagreements. Spearman’s rank correlation coefficient (r_s_) was reported as a supplementary measure of association, which, unlike κ, is insensitive to systematic shifts between raters. The 95% confidence intervals (CIs) of all κ and r_s_ values were estimated by nonparametric cluster bootstrap resampling at the patient level [45]. As a sensitivity analysis, unweighted κ for the TI-RADS categories and management recommendations was recalculated in 500 random subsamples comprising one TN per patient. A *p*-value < 0.05 was considered significant. Given the exploratory character of the feature-level comparisons, no adjustment for multiple testing was applied.

Spearman’s correlation coefficients were labeled “weak” for values ranging from 0.200 to 0.299, “moderate” for values ranging from 0.300 to 0.399, “strong” for values ranging from 0.400 to 0.699, and “very strong” for correlations > 0.700 [46].

According to Landis and Koch, κ < 0 shows poor agreement, whereas values of κ = 0.00 to 0.20 represent slight agreement, κ = 0.21 to 0.40 fair agreement, κ = 0.41 to 0.60 moderate agreement, κ = 0.61 to 0.80 substantial agreement, and κ = 0.81 to 1.00 almost perfect agreement [47].

## 3. Results

### 3.1. Patient Data and Clinical Characteristics

A total of 276 TNs in 118 consecutive patients were included (36 males, 30.5%; 82 females, 69.5%; range 15–86 years, mean age: 60.4 ± 15.4 years). A total of 36 (30.5%) patients had one TN, 31 (26.3%) two TNs and 51 (43.2%) three or more TNs. Thus, 82 patients (69.5%) contributed two or more TNs, accounting for 240 of 276 TNs (87.0%).

TNs were located in the right lobe in 121 (43.8%), in the left lobe in 122 (44.2%), and in the isthmus in 33 cases (12.0%). The diameters of the included TNs ranged from 4.1 to 66.1 mm (mean: 19.3 ± 10.6 mm). Of these TNs, 53 had a diameter of ≤10 mm (19.2%).

No malignancy was identified in any TN during follow-up. Of the benign TNs, 3 (1.1%) TNs were confirmed histologically following surgical resection, and 3 (1.1%) TNs were diagnosed cytologically. Furthermore, 64 (23.2%) TNs were diagnosed by scintigraphy as AFTNs. The remaining 206 cases (74.6%) were stable on ultrasound follow-up.

### 3.2. Ultrasound Features

Table 2 presents the distribution of ultrasound features along with the corresponding statistical analyses, comprising a comparison between the assessments of the expert panel and the DS System under investigation.

**Figure 1:**
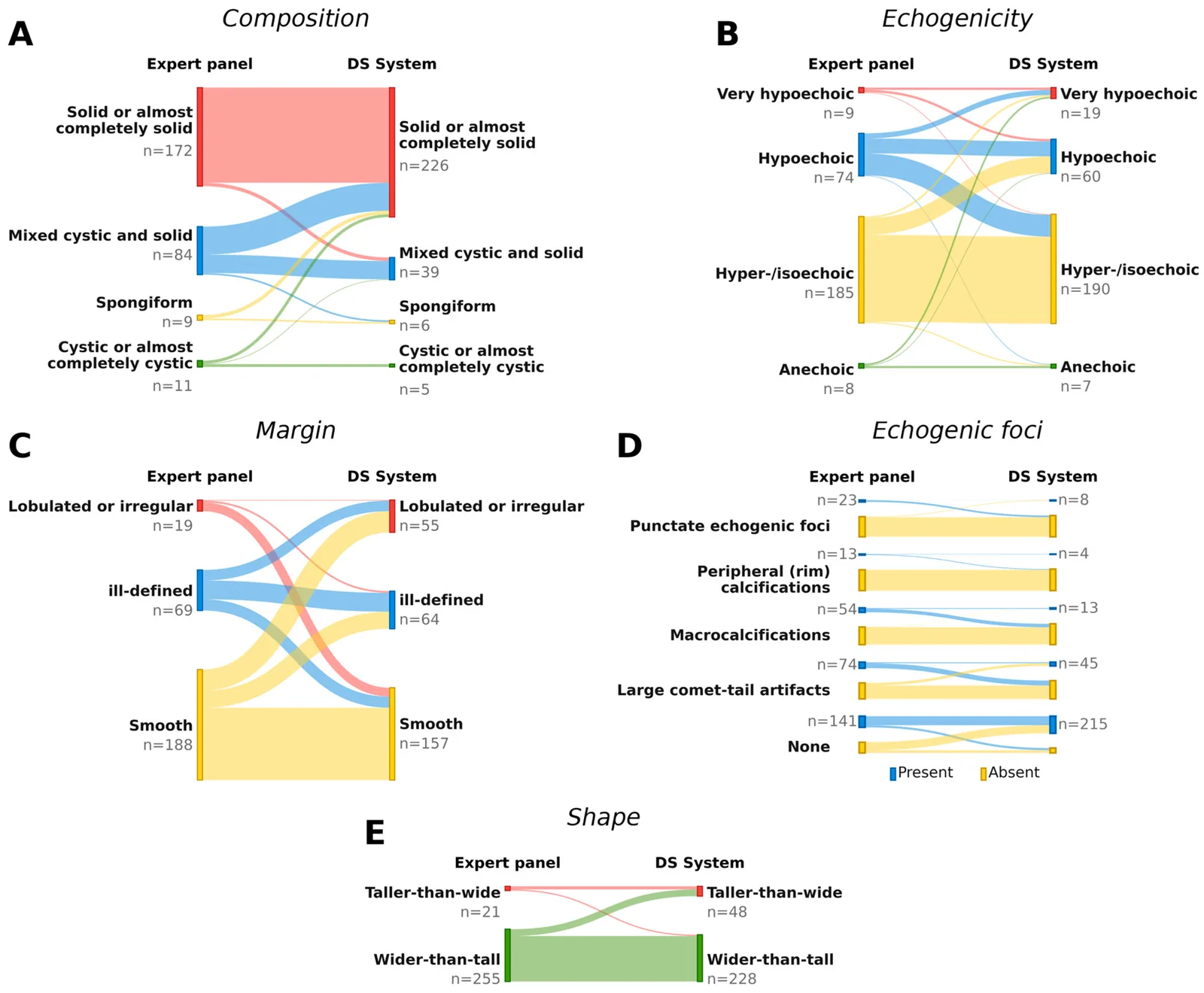
Ultrasound feature shifts (Sankey diagrams, n = 276). (**A**) Composition; (**B**) echogenicity; (**C**) margin; (**D**) echogenic foci; (**E**) shape.

With the exception of echogenicity, the marginal distributions of all feature categories differed significantly between the expert panel and the DS System (Table 2). Cohen’s κ values ranged between −0.08 (lobulated or irregular margins) and 0.62 (cystic or almost completely cystic), corresponding to poor to substantial interrater agreement between the expert panel and the DS System. Further details of the assessment differences are displayed in the Sankey flow diagrams in Figure 1.

In the *composition* category, the marginal distributions differed significantly (Table 2), with moderate agreement (κ = 0.43). The DS System classified the TNs considerably more often as solid or almost completely solid (226 vs. 172) and less often as mixed cystic and solid (39 vs. 84) than the expert panel. Of the 84 TNs classified as mixed cystic and solid by the expert panel, the DS System classified 49 (58.3% of 84) of these as solid or almost completely solid (Figure 1A). Six of nine TNs evaluated as spongiform by the expert panel were classified as solid or almost completely solid by the DS System. In contrast, half of the TNs classified as spongiform by the DS System (three out of six) were classified as mixed cystic and solid by the expert panel.

In the *echogenicity* category, the marginal distributions did not differ significantly (*p* = 0.23), although agreement at the level of the individual TN was only fair (κ = 0.31; identical ratings in 185 of 276 TNs, 67.0%). This apparent discrepancy results from bidirectional and largely balanced discordances, which offset each other in the marginal totals: 38 TNs rated hypoechoic by the expert panel were rated hyperechoic or isoechoic by the DS System, whereas 29 TNs were reclassified in the opposite direction. Consequently, the net differences in the category frequencies remained small (e.g., hypoechoic 74 vs. 60; hyperechoic or isoechoic 185 vs. 190), and tests of marginal homogeneity are, by design, insensitive to such offsetting discordances at the level of the individual TN. At the subcategory level, only the very hypoechoic subcategory was assigned more frequently by the DS System (19 vs. 9; *p* = 0.05). Of the 74 TNs classified as hypoechoic by the expert panel, the DS System classified 26 (35.1% of 74) in the same category, whereas 38 (51.4% of 74) were classified as hyperechoic or isoechoic, nine (12.2% of 74) as very hypoechoic and one (1.4% of 74) as anechoic (Figure 1B). Conversely, 29 (15.7% of 185) of the TNs classified as hyperechoic or isoechoic by the expert panel were classified as hypoechoic by the DS System, and three (1.6% of 185) were classified as very hypoechoic. Of the 19 TNs classified as very hypoechoic by the DS System, four (21.1% of 19) had been classified as very hypoechoic by the expert panel, nine (47.4% of 19) as hypoechoic and three (15.8% of 19) as anechoic.

In the *margin* category, the marginal distributions differed significantly (Table 2), with slight agreement (κ = 0.20). The DS System classified the TNs more frequently as lobulated or irregular (55 vs. 19) and less frequently as smooth (157 vs. 188). Extrathyroidal extension was not assigned by either rater. For lobulated or irregular, there was poor agreement (κ = −0.08). Of the 19 TNs classified as lobulated or irregular by the expert panel, the DS System classified one (5.3% of 19) in the same category, whereas 15 (78.9% of 19) were classified as smooth and three (15.8% of 19) as ill-defined (Figure 1C). Of the 55 TNs classified as lobulated or irregular by the DS System, 36 (65.5% of 55) had been classified as smooth by the expert panel and 18 (32.7% of 55) as ill-defined.

In the *echogenic foci* category, the DS System assigned the subcategory “none” more often than the expert panel (215 vs. 141), whereas all other subcategories were recorded less frequently (Table 2). The expert panel and DS System both classified punctate echogenic foci as present in 1 TN, peripheral (rim) calcifications in 4 TNs, and macrocalcifications in 10 TNs (Figure 1D).

In the *shape* category, the marginal distributions differed significantly (Table 2), with fair agreement (κ = 0.37). The DS System classified the TNs more frequently as taller-than-wide (48 vs. 21). Of the 21 TNs classified as taller-than-wide by the expert panel, the DS System classified 15 (71.4% of 21) in the same category, whereas six (28.6% of 21) were classified as wider-than-tall (Figure 1E). Of the 48 TNs classified as taller-than-wide by the DS System, 33 (68.8% of 48) had been classified as wider-than-tall by the expert panel.

### 3.3. ACR TI-RADS (Without AI-Adapter)

Table 3 presents the distribution of the ACR TI-RADS categories and the derived management recommendations, alongside the corresponding statistical analyses consisting of a comparison between the assessments of the expert panel and the DS System without AI-Adapter.

**Figure 2:**
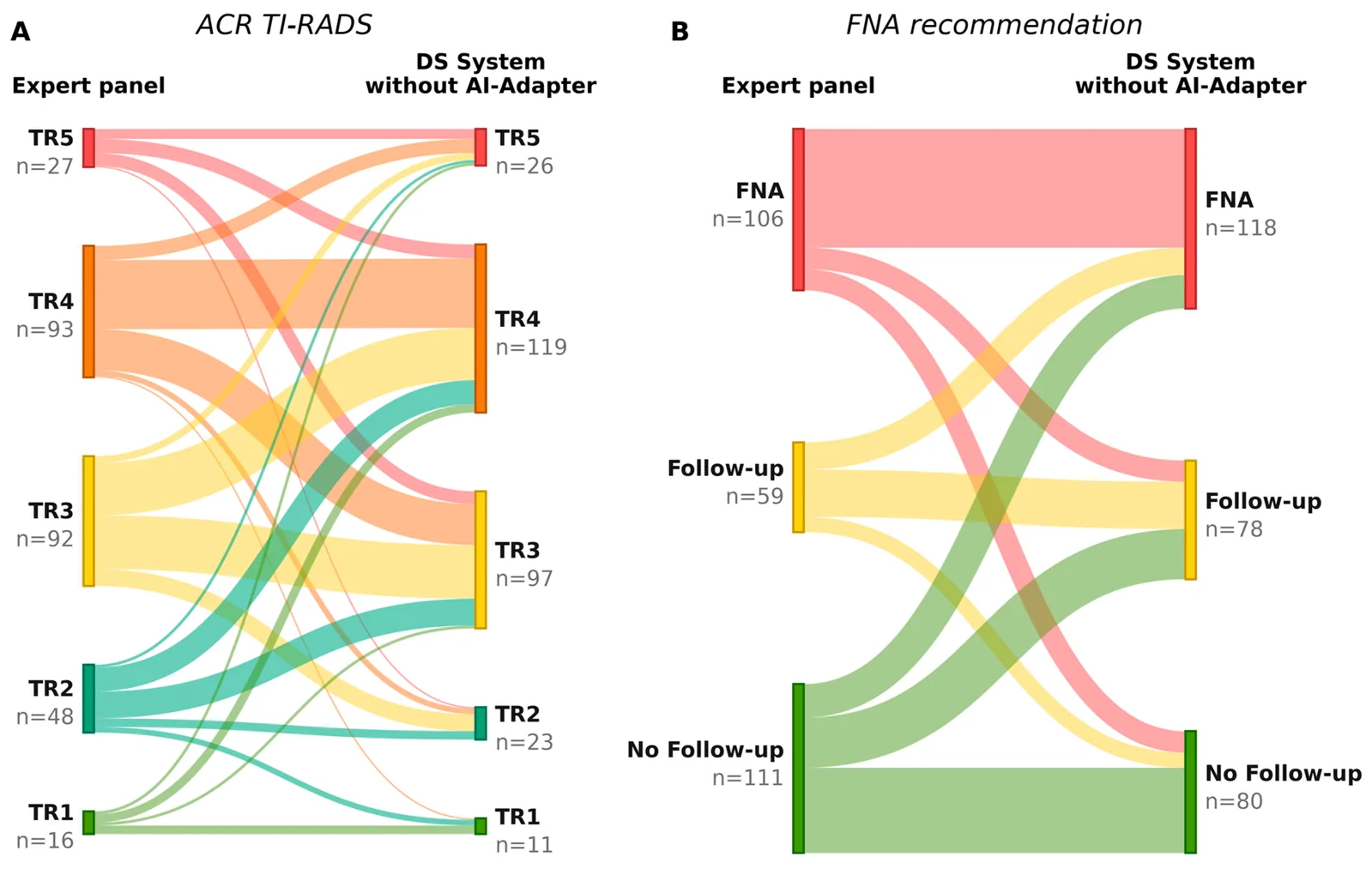
(**A**) TR-Rating and (**B**) FNA recommendation shift on expert panel vs. DS System without AI-Adapter.

The marginal distributions differed between the expert panel and the DS System without AI-Adapter for both the ACR TI-RADS rating (*p* = 0.01) and the recommendation (*p* < 0.01), with slight agreement for the RSS rating (κ = 0.13; linear-weighted κ = 0.21, 95% CI 0.12–0.30; quadratic-weighted κ = 0.28) and fair agreement for the recommendation (κ = 0.39; linear-weighted κ = 0.43, 95% CI 0.33–0.53; quadratic-weighted κ = 0.47). The Sankey flow diagrams in Figure 2 illustrate the assessment variations in further detail.

The DS System assigned TR4 more frequently (119 vs. 93) and TR2 less frequently (23 vs. 48) than the expert panel, whereas the frequencies of TR1 (11 vs. 16), TR3 (97 vs. 92) and TR5 (26 vs. 27) did not differ significantly (Table 3). For the individual TR classes, the range went from slight (TR2: κ = 0.06; TR3: κ = 0.09; TR4: κ = 0.14; TR5: κ = 0.19) to moderate (TR1: κ = 0.42). Of the 26 TNs classified as TR5 by the DS System, 19 (73.1% of 26) had been assigned to a lower category by the expert panel, comprising 10 TNs classified as TR4 (10.8% of 93), five as TR3 (5.4% of 92), two as TR2 (4.2% of 48), and two as TR1 (12.5% of 16) (Figure 2A). Conversely, of the 27 TNs classified as TR5 by the expert panel, the DS System classified seven (25.9% of 27) in the same category, whereas 10 (37.0% of 27) were classified as TR4, nine (33.3% of 27) as TR3, and one (3.7% of 27) as TR2. Thus, despite almost identical TR5 frequencies, only 7 TNs were rated TR5 by both raters, corresponding to 25.9% of the TR5 ratings of the expert panel and 26.9% of those of the DS System. The DS System recommended no follow-up less frequently (80 vs. 111) and follow-up more frequently (78 vs. 59) than the expert panel, whereas the frequency of FNA recommendations did not differ significantly (118 vs. 106) (Table 3). The range of agreement went from fair (follow-up: κ = 0.28; no follow-up: κ = 0.38) to moderate (FNA: κ = 0.49). Of the 118 TNs for which the DS System recommended FNA, 78 (66.1% of 118) had also been assigned to FNA by the expert panel, whereas 40 (33.9% of 118) had received a less invasive recommendation (Figure 2B). Conversely, of the 106 TNs for which the expert panel recommended FNA, the DS System issued the same recommendation in 78 TNs (73.6% of 106), whereas 28 (26.4% of 106) received a less invasive recommendation.

### 3.4. ACR TI-RADS (with AI-Adapter)

Table 4 presents the comparison between the expert panel and the DS System with AI-Adapter. The marginal distributions differed for both the RSS rating (*p* < 0.001) and the recommendation (*p* < 0.001).

**Figure 3:**
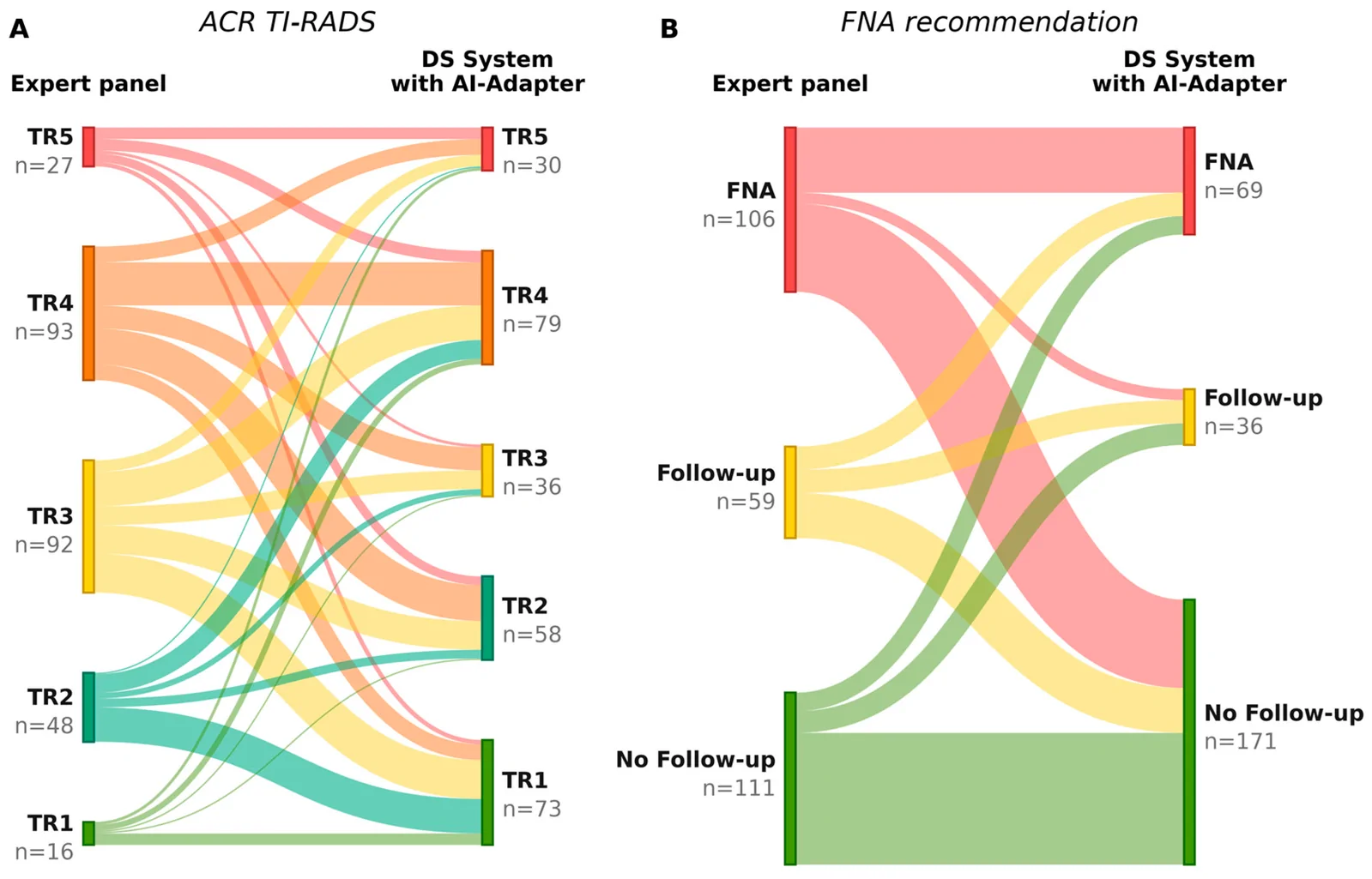
(**A**) TR-Rating and (**B**) FNA recommendation shift on expert panel vs. DS System with AI-Adapter.

The RSS rating exhibited slight agreement (κ = 0.04; linear-weighted κ = 0.14, 95% CI 0.07–0.20; quadratic-weighted κ = 0.23), while the recommendation demonstrated fair agreement (κ = 0.23; linear-weighted κ = 0.24, 95% CI 0.15–0.32; quadratic-weighted κ = 0.25). In the sensitivity analysis restricted to one randomly selected TN per patient, the median κ values were 0.06 (without) and 0.02 (with AI-Adapter) for the TI-RADS categories and 0.35 and 0.19 for the recommendations, respectively, and thus fell within the 95% CIs of the primary analysis. Figure 3 illustrates the assessment variations in this comparison.

The DS System assigned TR1 more often (73 vs. 16) and TR3 less often (36 vs. 92) than the expert panel, whereas the frequencies of TR2 (58 vs. 48), TR4 (79 vs. 93) and TR5 (30 vs. 27) did not differ significantly (Table 4). The range for the individual TR classes went from poor (TR2: κ = −0.10) to slight (TR3: κ = 0.02; TR4: κ = 0.06; TR1: κ = 0.09; TR5: κ = 0.20). Of the 30 TNs classified as TR5 by the DS System, 22 (73.3% of 30) had been assigned to a lower category by the expert panel, comprising 11 TNs classified as TR4 (11.8% of 93), eight as TR3 (8.7% of 92), two as TR1 (12.5% of 16), and one as TR2 (2.1% of 48) (Figure 3A). Conversely, of the 27 TNs classified as TR5 by the expert panel, the DS System classified eight (29.6% of 27) in the same category, whereas eight (29.6% of 27) were classified as TR4, six (22.2% of 27) as TR2, 3 (11.1% of 27) as TR1 and two (7.4% of 27) as TR3. Thus, only eight TNs were rated TR5 by both raters (29.6% of the TR5 ratings of the expert panel and 26.7% of those of the DS System), and nine of the 27 TNs rated TR5 by the expert panel (33.3%) were assigned to TR1 or TR2 by the DS System with AI-Adapter. The DS System recommended no follow-up more frequently (171 vs. 111) and both follow-up (36 vs. 59) and FNA (69 vs. 106) less frequently than the expert panel (Table 4), while the range of agreement went from slight (follow-up: κ = 0.18) to fair (no follow-up: κ = 0.22; FNA: κ = 0.25). Of the 69 TNs for which the DS System recommended FNA, 42 (60.9% of 69) had also been assigned to FNA by the expert panel, whereas 27 (39.1% of 69) had received a less invasive recommendation (Figure 3B). Conversely, of the 106 TNs for which the expert panel recommended FNA, the DS System issued the same recommendation in 42 (39.6% of 106), whereas 64 (60.4% of 106) received a less invasive recommendation.

**Figure 4:**
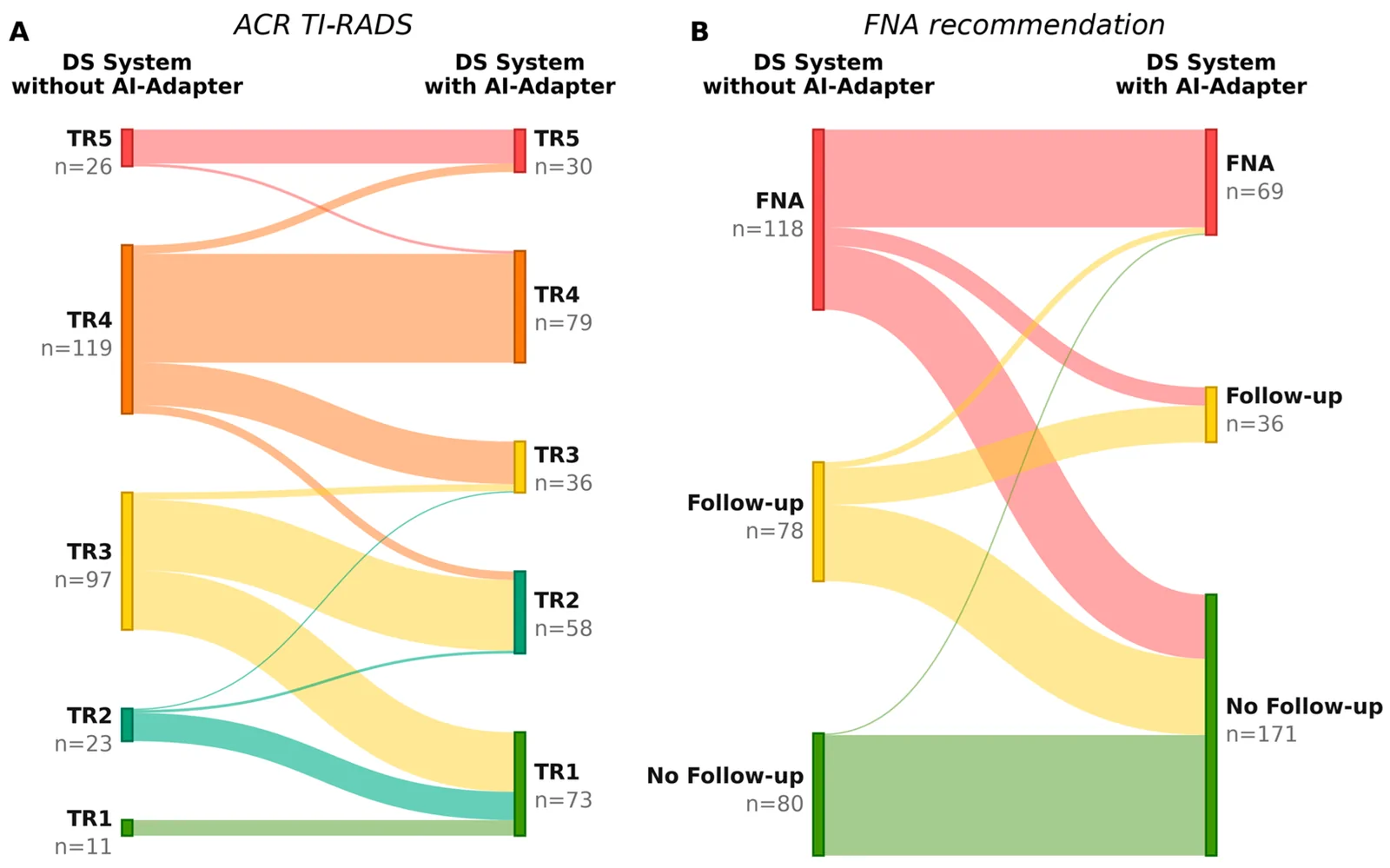
(**A**) TR-Rating and (**B**) FNA recommendation shift on DS System without vs. with AI-Adapter.

The AI-Adapter changed the ACR TI-RADS score by a mean of −1.01 ± 1.04 points (median −1.0, range −2.0 to +2.0). The resulting distribution of the TR categories differed from that obtained without the AI-Adapter (*p* < 0.001). The shift induced by the AI-Adapter mainly affected the intermediate categories, whereas slightly more TNs were assigned to TR5 with than without the AI-Adapter (n = 30 vs. n = 26) (Figure 4A).

The distribution of the derived recommendations likewise differed (*p* < 0.001). The AI-Adapter resulted in a decrease in TNs assigned to FNA (n = 118 vs. n = 69) and follow-up (n = 78 vs. n = 36) but an increase in no follow-up recommendations (n = 80 vs. n = 171) (Figure 4B).

All 27 TNs that were classified as TR5 by the expert panel were benign according to the composite reference standard, as confirmed by histological examination in three cases (11.1% of 27) and by cytological examination in three cases (11.1% of 27). AFTNs were diagnosed in 16 TNs (59.3% of 27), and the remaining fiveTNs (18.5% of 27) were stable on ultrasound follow-up, which was performed instead of FNA at the patient’s request.

## 4. Discussion

### 4.1. Low Levels of Agreement Between Expert Panel and DS System

The findings of this study indicate substantial discrepancies between the assessments of the expert panel and the DS System for all investigated parameters: ultrasound features (except for echogenicity), ACR TI-RADS classifications and FNA recommendations. The low levels of agreement are not confined to a single feature category but extend across nearly all categories (κ ranging from −0.10 to 0.62; slight and fair agreement was most commonly seen). Given the additive composition of the ACR TI-RADS classification, the disparity in ultrasound features is directly transferred to the subsequent risk stratification (TI-RADS classification). Weak correlations were found for ACR TI-RADS (r_s_ = 0.27 with and r_s_ = 0.24 without the AI-Adapter). Management recommendations showed weak (r_s_ = 0.25 with AI-Adapter) and strong correlations (r_s_ = 0.49 without AI-Adapter). Linear-weighted κ, which gives partial credit to disagreements between adjacent categories, was 0.21 without and 0.14 with AI-Adapter for the TI-RADS classification and thus reached at most the lower limit of fair agreement.

**Figure 5:**
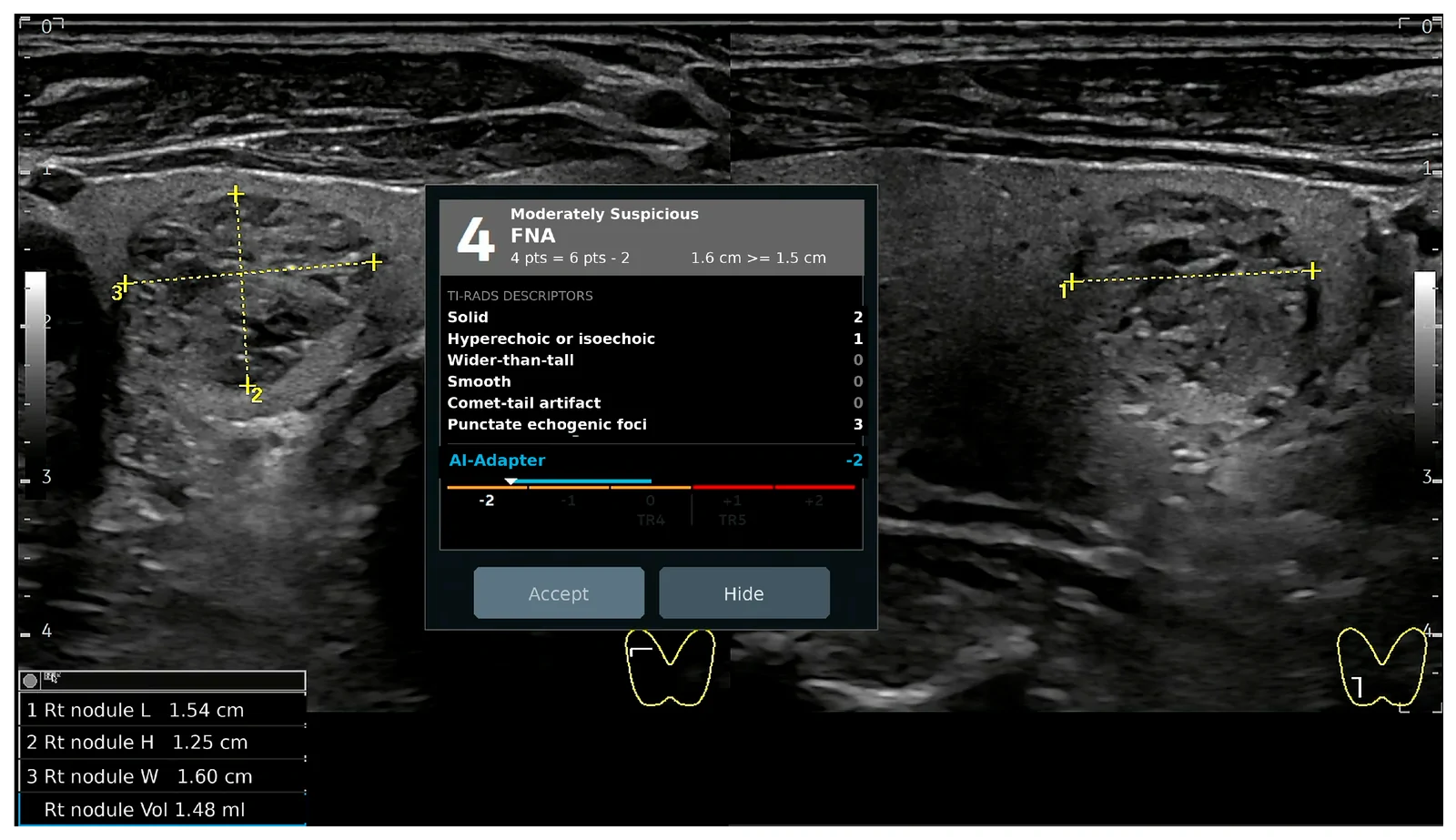
Example of a spongiform TN without punctate echogenic foci interpreted as solid with punctate echogenic foci by the DS System (resulting in a TR4 rating). In this case, the AI-Adapter decreased the score by 2 points.

The composition category exhibited a salient shift in the assessment of individual TNs between the expert panel and the DS System. Notably, TNs classified as mixed cystic and solid by the expert panel were frequently rated as solid or almost completely solid by the DS System, corresponding to an increase of 1 point in the ACR TI-RADS. This phenomenon is further illustrated by the assessment of spongiform TNs, wherein the DS System and the expert panel employed divergent classifications for the majority of cases. The honeycomb-like internal architecture, characterized by multiple small cystic areas, appeared not to be recognized as such by the DS System in several cases. Instead, these TNs were classified as solid, as illustrated in Figure 5. As the internal decision process of the DS System is not accessible, this observation describes the output pattern rather than its underlying mechanism. This is of particular clinical interest because the ACR TI-RADS RSS assigns a score of 0 points to lesions classified as spongiform. This is due to the fact that spongiform TNs are almost always benign [38,48]. Misclassification as solid adds 2 points.

**Figure 6:**
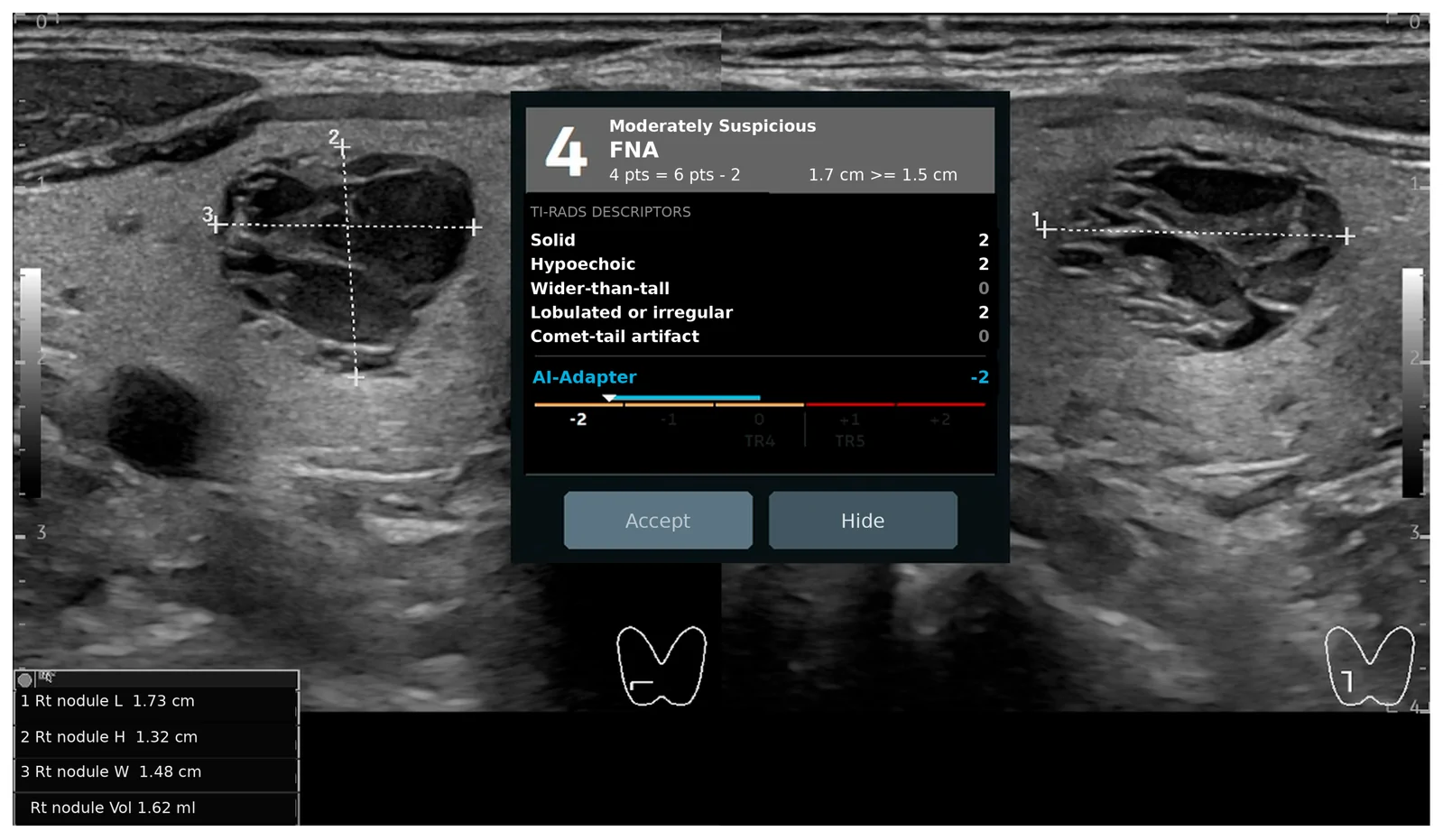
Example of a mixed cystic and solid TN interpreted as solid and hypoechoic by the DS System (resulting in a TR4 rating). In this case, the AI-Adapter decreased the score by 2 points.

For echogenicity, the marginal distributions did not differ significantly, whereas the category-specific agreement at the level of the individual TN was only slight to moderate. As shown in Section 3.2, this reflects bidirectional discordances that offset each other in the marginal totals. Similar marginal distributions therefore do not imply concordant ratings of individual TNs. A considerable proportion of TNs that were classified as hyperechoic or isoechoic by the expert panel were assessed as hypoechoic by the DS System. On retrospective visual review, these included TNs with cystic components, which may have lowered the overall echogenicity assigned by the DS System, as illustrated in Figure 6.

One hypothesis for the bidirectional variation between “hypoechoic” and “hyperechoic or isoechoic” is that the DS System may rely on absolute gray values or on a reference region other than the adjacent thyroid parenchyma, relative to which echogenicity is defined in ACR TI-RADS [16]. Such a difference in the reference would be expected to result in deviations in both directions, as observed in the data. This hypothesis cannot be tested with the present data, as the internal decision process of the DS System is not disclosed. If confirmed, it would be of particular relevance in populations with a heterogeneous background parenchyma, such as in nodular goiter or autoimmune thyroiditis.

The lowest level of agreement in this study was observed for the ultrasound feature of margin (slight, κ = 0.20). Discrepancies occurred in both directions. This finding is particularly noteworthy for the lobulated or irregular subcategory, where both raters assigned the same rating to only one TN and provided different ratings in all other cases.

The distinction between punctate echogenic foci and reflections related to artifacts (such as comet-tail artifacts) is conceptually challenging. The discrepancies between the DS System and the expert panel for this category are consistent with limited reproducibility of this feature; whether they reflect limitations of automated detection or the inherent ambiguity of the feature cannot be distinguished with the present data.

As shown in Figure 1E, the expert panel and the DS System consistently classified the vast majority of TNs as wider-than-tall. Nevertheless, κ was 0.37 (fair agreement). This discrepancy may be attributed to the highly uneven distribution. Since the expert panel classified 92.4% of TNs as wider-than-tall, a high proportion of matching ratings is expected by chance alone. This leaves only a narrow margin for a chance-corrected agreement. This phenomenon, known as the Kappa paradox, restricts the comparability of Kappa values between categories with different prevalence distributions [49]. Beyond this statistical effect, however, the DS System assigned taller-than-wide more than twice as often as the expert panel (48 vs. 21), which is clinically relevant given the weight of 3 points for this feature.

The practical relevance of the discrepancies observed is contingent upon the differential weighting of individual ultrasound features within the ACR TI-RADS. The highest point value (3 points each) applies to very hypoechoic, taller-than-wide, punctate echogenic foci, and extrathyroidal extension. Hypoechoic, lobulated or irregular, and peripheral (rim) calcifications contribute 2 points each [16]. Deviations in these criteria have a substantial effect on the resulting TR classification, while discrepancies in ultrasound features of low impact leave the overall classification unchanged. Agreement was low for several of these highly weighted features (e.g., hypoechoic: κ = 0.19; very hypoechoic: κ = 0.25; lobulated or irregular: κ = −0.08; punctate echogenic foci: κ = 0.02). Given that a divergent assessment among these ultrasound features has the potential to shift a TN across the threshold between two TR categories, this pattern may explain why the differences observed at the ultrasound feature level translate into a markedly divergent TR classification distribution.

A further aspect to consider is that AFTNs frequently reach high TR classifications [39,40]. AFTNs were identified in 64 TNs (23.2% of 276), indicating that a significant proportion of this cohort may appear sonographically suspicious, despite being presumably benign. In our data, 16 AFTNs were rated as TR5 by the expert panel. Deviations in the highly weighted features therefore occur in a setting in which high TR categories are already prevalent for reasons unrelated to malignancy risk.

**Figure 7:**
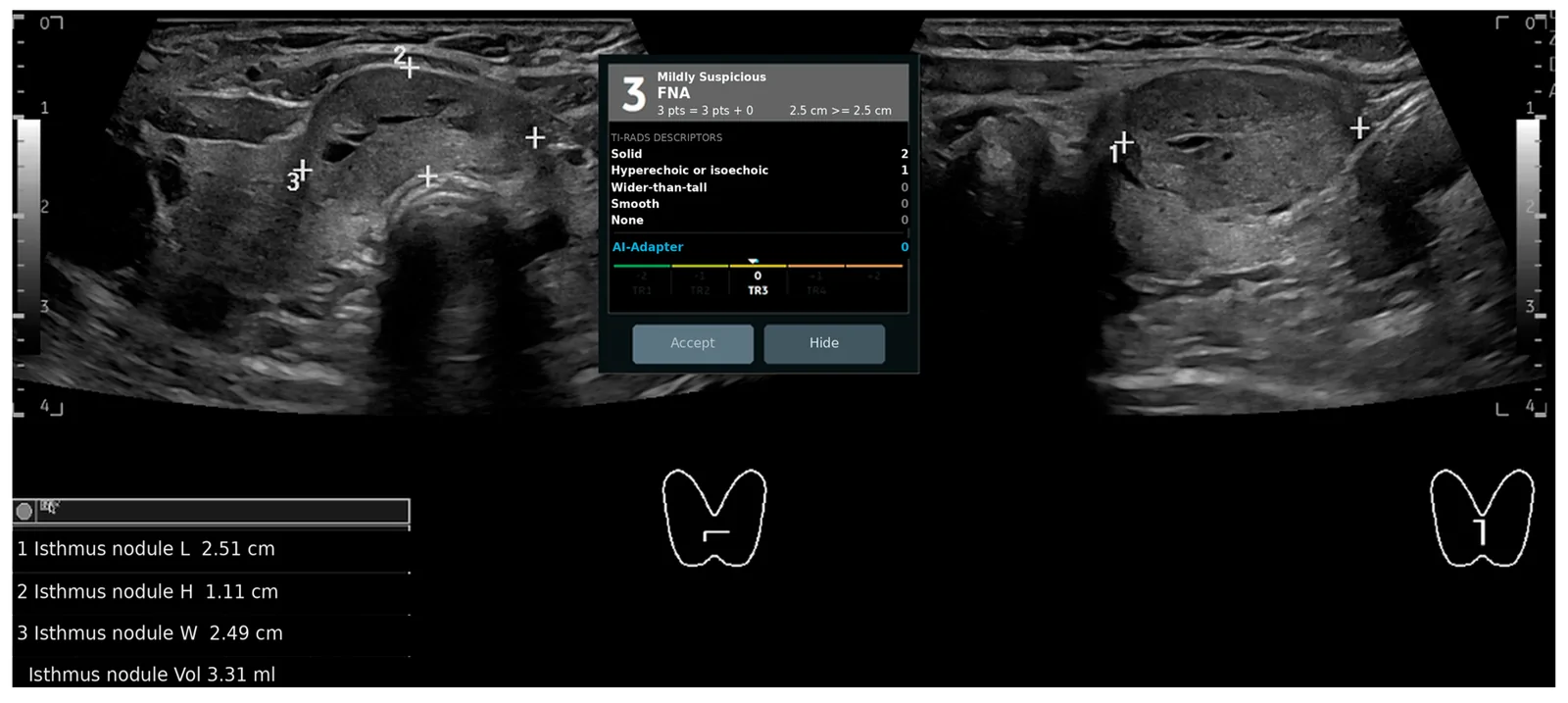
Example of a TN with identical interpretation by expert panel and DS System; no AI-Adapter influence.

Despite the emphasis on the discrepancies, it should be noted that a substantial portion of the TNs were accorded similar ratings by the expert panel and the DS System. The flow diagrams show broad, non-overlapping bands for several categories—for example, solid TNs, TNs with a hyperechoic or isoechoic structure or TNs classified as wider-than-tall. Qualitatively, agreement appeared better when morphological criteria were clearly defined and no borderline decisions were needed (Figure 7); this impression was not quantified.

### 4.2. Effect of the AI-Adapter

The AI-Adapter produced a directional downgrading of the DS System-based assessment, affecting primarily TNs in the intermediate categories TR3 and TR4. In the entire cohort, the mean change was −1.01 ± 1.04 points (median: −1.0, range: −2.0 to 2.0). Because all TNs in this cohort were benign on follow-up, this downward shift is directionally consistent with the reference standard; however, it cannot be determined whether malignant TNs would be downgraded to a similar extent. Moreover, the AI-Adapter does not shift the entire scale uniformly. TNs with a low to intermediate risk profile are downgraded, especially TR3 and TR4, while a few TNs with a high TR classification were further upgraded. Consequently, a greater proportion of TNs were classified as TR5 with the AI-Adapter (n = 30; 10.9%) than without it (n = 26; 9.4%). The downgrading described is directly reflected in the derived management recommendations. The DS System without AI-Adapter recommended FNA for 118 TNs (42.8%), which exceeded the expert panel evaluation (n = 106; 38.4%). With AI-Adapter, this number substantially fell to 69 TNs (25.0%). The AI-Adapter, therefore, does not merely modify the recommendation practice in relation to the DS System without the AI-Adapter, but also results in a substantially more conservative determination of indications in comparison to the expert panel assessment. A reduction in FNA recommendations by the DS System or comparable machine-learning approaches has also been reported in cohorts with histological or cytological reference standards [19,30,50]. In the present cohort, however, this reduction cannot be equated with a reduction in unnecessary FNAs, because the absence of malignant TNs precludes any assessment of whether suspicious TNs would have been inappropriately downgraded. The AI-Adapter did not increase agreement with the expert panel assessment (κ = 0.04 vs. κ = 0.13). Consequently, rather than facilitating a closer alignment between the DS System and the expert panel assessment, it results in a systematically more conservative classification. Whether this constitutes an improvement cannot be determined without an independent reference standard, given the potential divergence between agreement and diagnostic accuracy, as evidenced in a pediatric cohort by higher sensitivity concomitant with lower specificity [51].

The TR5 category deserves particular attention, as it represents the highest-risk category and triggers FNA at the lowest size threshold [16]. At the aggregate level, the DS System reproduced the frequency of TR5 ratings almost exactly (expert panel: n = 27, 9.8%; DS System without AI-Adapter: n = 26, 9.4%, *p* = 0.87; with AI-Adapter: n = 30, 10.9%, *p* = 0.64). This indicates that the proportion of TNs regarded as highly suspicious was similar at the population level, which is a relevant prerequisite for clinical use, as a systematic over- or underestimation of the highest-risk category would directly translate into systematically higher or lower FNA rates. At the level of the individual TN, however, concordance was low: only 7 (without AI-Adapter) and 8 (with AI-Adapter) of the 27 TR5 TNs of the expert panel were also rated TR5 by the DS System (κ = 0.19 and κ = 0.20, respectively), whereas approximately three quarters of the TR5 ratings of the DS System concerned TNs that the expert panel had assigned to lower categories. With AI-Adapter, 9 of the 27 TR5 TNs of the expert panel (33.3%) were assigned to TR1 or TR2, i.e., to categories for which ACR TI-RADS recommends neither FNA nor follow-up irrespective of size [16]. The similarity of the TR5 frequencies thus results from two opposing and largely offsetting discordances rather than from concordant classification and must not be interpreted as evidence of a reliable identification of high-risk TNs. As all TNs in this cohort were benign, the clinical consequences of this discordance for malignant TNs cannot be assessed.

### 4.3. Study Results in Relation to the Existing Literature

The limited levels of agreement observed in this study are consistent with those previously documented in human interrater data [52]. A multicenter study involving seven radiologists and endocrinologists reported only fair agreements (for example regarding margins with κ = 0.33) [53]. A comparison between two blinded raters reached only slight agreement (κ = 0.14) [54]. In another study with four experienced raters, the initial Fleiss κ for ACR TI-RADS was 0.32 and increased to 0.57 after consensus reading sessions, without improvement in diagnostic accuracy [55]. The values determined in this study fall below this level, which seems plausible, since the comparison here was not between two human evaluators with similar backgrounds, but rather between fundamentally different evaluation principles. The limited agreement is also consistent with the understanding that the performance of AI-assisted systems is profoundly influenced by the specific nature of the application context. In one study, a comparative analysis of the internal test dataset revealed that deep learning models demonstrated a marked superiority over the performance of radiologists. However, this advantage was not replicated in other datasets [56]. Similarly, an elevated error rate was documented for TNs measuring ≤10 millimeters in a prospectively evaluated AI system [57]. This observation underscores the constrained transferability to distinct cohorts. For the DS System evaluated in the present study, independent external data have recently become available. In a cohort of histologically confirmed TNs, the AI-based reclassification shifted 81.4% of ACR TI-RADS 3 and 24.5% of TI-RADS 4 TNs into lower risk categories, consistent with the predominant downgrading of intermediate categories observed in this study [31]. Moreover, only fair agreement (κ = 0.25) between automated and radiologist-derived TI-RADS levels was reported for cytologically indeterminate TNs [36], and pronounced feature-level discrepancies regarding composition and echogenicity were also described in a non-subspecialist setting [35]. Overall, our results show a lower degree of agreement in comparison to previously published data. However, these studies were conducted in regions with substantially lower prevalence of TNs and therefore in differently composed cohorts, which limits the comparability of the results [1]. Whether the lower agreement observed here is attributable to population characteristics associated with historical iodine deficiency, to the real-time application at the ultrasound console, or to other factors, such as the reference panel, equipment or case mix, cannot be determined from the present single-population study without a comparison cohort. This explanation therefore remains a hypothesis. A summary of previous studies on the DS System under investigation is presented in Table 5. A further major distinction between these studies is that the DS System was retrospectively implemented on a PACS workstation, whereas our data were prospectively acquired in real-time on the ultrasound device.

### 4.4. Outlook

**Figure 8:**
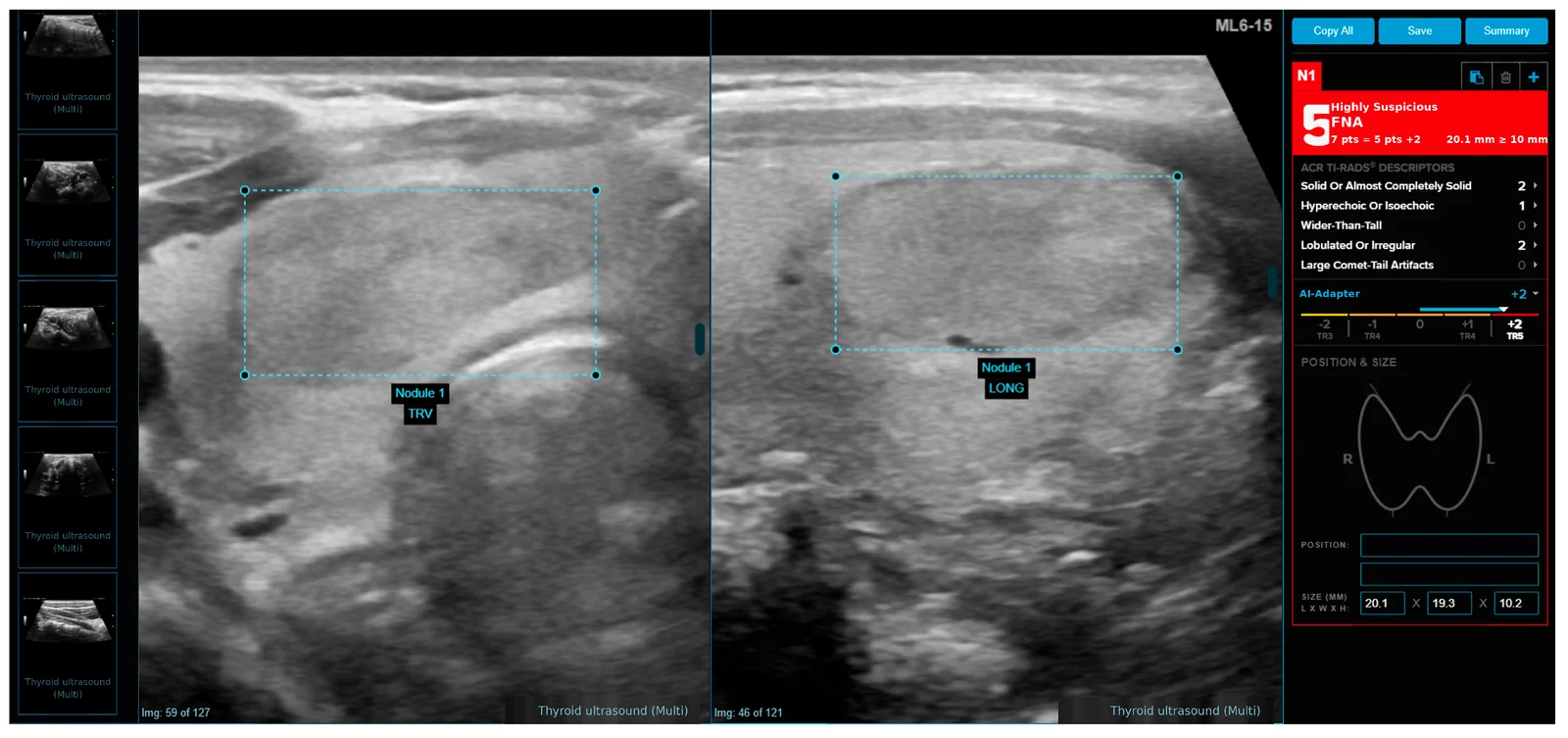
Illustrative example of the DS System applied to ultrasound cine-loop data on a PACS workstation (technical illustration only).

In addition to the use of the DS System on the ultrasound console, it can also be applied afterwards on the DICOM image data archived in the local PACS (Figure 8). This enables the application of the DS System to ultrasound video sequences (so-called cine loops), thereby facilitating the re-evaluation of the most critical parts of the TNs that may have been overlooked during the live examination [58,59]. The technical feasibility of this approach was confirmed in our PACS environment (GE HealthCare). A systematic evaluation of this approach was beyond the scope of the present study and appears to be of special interest for future research.

### 4.5. Limitations

The most important limitation of this study is that no malignant TN was identified. This does not result from a selection of benign TNs, as all consecutive patients were included regardless of the sonographic or functional characteristics of their TNs, but from the low pre-test probability of malignancy in this referral population from a formerly iodine-deficient region. Nevertheless, the resulting case mix is extremely unbalanced with respect to the target condition and constitutes a spectrum effect that limits the generalizability of the findings [60]. Consequently, the present study evaluates agreement with an expert reference assessment and not diagnostic performance. Sensitivity, negative predictive value and the safety of downgrading by the AI-Adapter, in particular the question of whether suspicious or malignant TNs would be inappropriately downgraded below the FNA threshold, cannot be estimated. Within this benign cohort, the proportions of TNs for which FNA was recommended (expert panel 38.4%; DS System 42.8% without and 25.0% with AI-Adapter) may at most be interpreted as apparent false-positive rates, conditional on the validity of the reference standard. Moreover, benignity was predominantly established by sonographic size stability over a 24-month period or by the scintigraphic detection of AFTNs and only in six TNs by cytological or histological confirmation, which constitutes an inferior and differential reference standard with partial verification. Although malignancy is rare in AFTNs and in TNs that remain stable over time [8,40], occasional malignancies may therefore have been missed. The findings do not allow any conclusions regarding the diagnostic accuracy or clinical safety of the DS System and need to be validated in cohorts that include histologically confirmed malignant TNs.

The expert panel assessment was established as a consensus reading of two experienced physicians without prior independent readings. Therefore, no inter-expert agreement data are available, and the variability of the reference assessment itself could not be quantified. Consensus reading has been shown to increase interobserver agreement, but not necessarily diagnostic accuracy [55]. In addition, the expert panel assessed images stored on a PACS workstation, whereas the DS System analyzed the images selected during the live examination; however, the same images were used.

Germany and Central Europe are regarded as regions with a documented history of iodine deficiency, characterized by a high prevalence of predominantly benign TNs [61,62]. Consequently, the size-dependent ACR recommendations for FNA (predominantly based on data from the U.S.) would lead to an unnecessarily high rate of FNAs in our region. In this context, most TNs for which FNA was recommended according to ACR TI-RADS were managed by scintigraphy and/or sonographic follow-up in accordance with the local standard of care, as agreed upon with the patients. Only 3 of the 106 TNs with an FNA recommendation by the expert panel underwent FNA. Finally, the assumption that population characteristics related to historical iodine deficiency contribute to the low agreement is plausible but speculative, as no comparison cohort from a region with different iodine supply was included. A multicenter comparison across regions is required to test this hypothesis.

The AI-Adapter is based on a proprietary, undisclosed algorithm. It was not possible to determine which input variables determine the magnitude of the correction factor. This fundamental limitation hinders the capacity to establish a causal connection between observed deviations and specific algorithmic properties. Furthermore, workflow-related parameters such as processing time, additional examination time, the number of repeated analyses and user burden were not systematically recorded. The observation that the DS System could be applied to all TNs without technical failure therefore only demonstrates technical feasibility and does not allow conclusions to be drawn about workflow efficiency.

No adjustment for multiple testing was applied. Given the exploratory character of the feature-level comparisons, *p*-values should be interpreted with caution. The clustering of TNs within patients was accounted for by cluster-robust tests and cluster bootstrap Cis. Nevertheless, the limited number of patients (n = 118) restricts the precision of the estimates, particularly for rare categories. Finally, given that the study was monocentric, it cannot be ruled out that the results may be influenced by the equipment used, the image acquisition process, and the members of the expert panel.

## 5. Conclusions

The findings of this study suggest that, when employed on a cohort with a high prevalence of predominantly benign TNs, the DS System evaluated cannot currently be regarded as a substitute for evaluation by experienced practitioners. The low levels of agreement with the ratings of an expert panel, particularly with regard to ultrasound features that impact the TR classifications, suggest that it should at most be regarded as a supplementary decision support instrument. Notably, the similar number of TR5 ratings concealed a low concordance at the level of the individual TN. As no malignant TNs were included, these results reflect agreement with expert assessment and do not allow conclusions regarding diagnostic accuracy or clinical safety. The real-time application on the ultrasound console with standardized documentation was technically feasible without failures, whereas its effect on workflow efficiency remains to be quantified. Additional prospective, multicenter studies are required in cohorts that include malignant TNs and reflect the regional variation in population composition. Furthermore, it would be beneficial to extend the applicability of the DS System to encompass additional ultrasound devices.

## Figures and Tables

**Table 1:** List of clinically implemented AI-based DS Systems for the risk stratification of TNs.

DS System	Status	Vendor	Application Options
S-Detect for Thyroid [27,28]	FDA-cleared; CE-marked	Samsung Medison (South Korea)	integrated on several Samsung ultrasound devices no workstation or PACS version
Koios DS Thyroid [29,30,31]	FDA-cleared; CE-marked	Koios Medical (USA)	can be integrated on GE LOGIQ E10 ultrasound device workstation/GE PACS integration for DICOM readout server-based, platform-agnostic web application
AmCAD-UT Detection [32,33]	FDA-cleared; no CE mark	AmCad BioMed (Taiwan)	no ultrasound device integration option standalone Windows workstation software
PIUR tUS Infinity [34]	FDA-cleared; CE-marked	PIUR Imaging (Austria)	evaluation on a separate Windows-based secondary hardware (PC) works with multiple different ultrasound systems no direct integration on the ultrasound console no integration into PACS workstations

**Table 2:** Ultrasound features: expert panel versus DS System.

Ultrasound Features	Expert Panel n (%)	DS System n (%)	*p* ^a^	κ (95% CI) ^b^	Interrater Agreement
**Composition**			**<0.001**	**0.43 (0.33–0.53)**	**moderate**
Solid or almost completely solid	172 (62.3)	226 (81.9)	<0.001	0.43 (0.33–0.53)	moderate
Mixed cystic and solid	84 (30.4)	39 (14.1)	<0.001	0.41 (0.28–0.53)	moderate
Cystic or almost completely cystic	11 (4.0)	5 (1.8)	0.03	0.62 (0.22–0.88)	substantial
Spongiform	9 (3.3)	6 (2.2)	0.32	0.38 (−0.01–0.61)	fair
**Echogenicity**			**0.23**	**0.31 (0.19–0.42)**	**fair**
Hypoechoic	74 (26.8)	60 (21.7)	0.16	0.19 (0.05–0.33)	slight
Very hypoechoic	9 (3.3)	19 (6.9)	0.05	0.25 (0.02–0.50)	fair
Hyperechoic or isoechoic	185 (67.0)	190 (68.8)	0.58	0.39 (0.26–0.52)	fair
Anechoic	8 (2.9)	7 (2.5)	0.71	0.52 (−0.01–0.77)	moderate
**Shape**			**<0.001**	**0.37 (0.23–0.51)**	**fair**
Taller-than-wide	21 (7.6)	48 (17.4)	<0.001	0.37 (0.22–0.51)	fair
Wider-than-tall	255 (92.4)	228 (82.6)	<0.001	0.37 (0.21–0.51)	fair
**Margin**			**<0.001**	**0.20 (0.11–0.28)**	**slight**
Smooth	188 (68.1)	157 (56.9)	<0.01	0.24 (0.13–0.35)	fair
Lobulated or irregular	19 (6.9)	55 (19.9)	<0.001	−0.08 (−0.14–−0.02)	poor
Ill-defined	69 (25.0)	64 (23.2)	0.59	0.32 (0.18–0.44)	fair
Extrathyroidal extension	0 (0.0)	0 (0.0)	-	-	no cases present
Echogenic Foci *			–		
None	141 (51.1)	215 (77.9)	<0.001	0.06 (−0.03–0.15)	slight
Large comet-tail artifacts	74 (26.8)	45 (16.3)	0.011	0.00 (−0.11–0.10)	poor
Macrocalcifications	54 (19.6)	13 (4.7)	<0.001	0.24 (0.10–0.37)	fair
Peripheral (rim) calcifications	13 (4.7)	4 (1.4)	<0.01	0.46 (0.00–0.74)	moderate
Punctate echogenic foci	23 (8.3)	8 (2.9)	<0.01	0.02 (−0.06–0.16)	slight

* Multiple subcategories may occur simultaneously; subcategories were therefore tested separately as binary variables. ^a^ Paired test of marginal homogeneity (Stuart–Maxwell test for feature categories, McNemar test for binary subcategories) with cluster-robust variance at patient level. ^b^ Cohen’s unweighted κ; 95% CI by patient-level cluster bootstrap.

**Table 3:** ACR TI-RADS: expert panel versus DS System without AI-Adapter.

	Expert Panel *n* (%)	DS System Without AI-Adapter *n* (%)	*p* ^a^	r_s_ (95% CI) ^b^	κ (95% CI) ^b^	Interrater Agreement
**ACR TI-RADS**			**0.01**	**0.24 (0.13–0.35)**	**0.13 (0.04–0.22)**	**slight**
Weighted κ (linear)					0.21 (0.12–0.30)	fair
TR1	16 (5.8)	11 (4.0)	0.20		0.42 (0.14–0.60)	moderate
TR2	48 (17.4)	23 (8.3)	<0.01		0.06 (−0.05–0.19)	slight
TR3	92 (33.3)	97 (35.1)	0.62		0.09 (−0.05–0.22)	slight
TR4	93 (33.7)	119 (43.1)	0.02		0.14 (0.02–0.24)	slight
TR5	27 (9.8)	26 (9.4)	0.86		0.19 (−0.01–0.38)	slight
**Recommendation**			**<0.01**	**0.49 (0.37–0.59)**	**0.39 (0.29–0.48)**	**fair**
Weighted κ (linear)					0.43 (0.33–0.53)	moderate
No Follow-up	111 (40.2)	80 (29.0)	<0.01		0.38 (0.26–0.48)	fair
Follow-up	59 (21.4)	78 (28.3)	0.02		0.28 (0.14–0.40)	fair
FNA	106 (38.4)	118 (42.8)	0.16		0.49 (0.36–0.60)	moderate

^a^ Paired test of marginal homogeneity (Stuart–Maxwell test for the overall distribution, McNemar test for individual categories) with cluster-robust variance at patient level. ^b^ 95% CI by patient-level cluster bootstrap. κ, Cohen’s unweighted kappa; weighted κ, linear-weighted kappa (quadratic-weighted κ is reported in the text); r_s_, Spearman’s rank correlation coefficient.

**Table 4:** ACR TI-RADS: expert panel versus DS System with AI-Adapter (n = 276).

	Expert Panel *n* (%)	DS System with AI-Adapter *n* (%)	*p* ^a^	r_s_ (95% CI) ^b^	κ (95% CI) ^b^	Interrater Agreement
**ACR TI-RADS**			**<0.001**	**0.27 (0.16–0.38)**	**0.04 (−0.02–0.10)**	**slight**
Weighted κ (linear)					0.14 (0.07–0.20)	slight
TR1	16 (5.8)	73 (26.4)	<0.001		0.09 (0.00–0.20)	slight
TR2	48 (17.4)	58 (21.0)	0.33		−0.10 (−0.20–0.02)	poor
TR3	92 (33.3)	36 (13.0)	<0.001		0.02 (−0.07–0.11)	slight
TR4	93 (33.7)	79 (28.6)	0.21		0.06 (−0.07–0.18)	slight
TR5	27 (9.8)	30 (10.9)	0.64		0.20 (0.01–0.39)	slight
**Recommendation**			**<0.001**	**0.25 (0.15–0.36)**	**0.23 (0.15–0.30)**	**fair**
Weighted κ (linear)					0.24 (0.15–0.32)	fair
No Follow-up	111 (40.2)	171 (62.0)	<0.001		0.22 (0.13–0.32)	fair
Follow-up	59 (21.4)	36 (13.0)	<0.01		0.18 (0.05–0.32)	slight
FNA	106 (38.4)	69 (25.0)	<0.001		0.25 (0.15–0.36)	fair

^a^ Paired test of marginal homogeneity (Stuart–Maxwell test for the overall distribution, McNemar test for individual categories) with cluster-robust variance at patient level. ^b^ 95% CI by patient-level cluster bootstrap. κ, Cohen’s unweighted kappa; weighted κ, linear-weighted kappa (quadratic-weighted κ is reported in the text); r_s_, Spearman’s rank correlation coefficient.

**Table 5:** Previous study reports on the application of the DS System under investigation.

Author, Year	Design	Patients	Nodules	Country	Main Results	Conclusion
**Barinov et al., 2023** [29]	retrospective, PACS workstation	650	650 (500 USA, 150 Spain)	USA & Spain	ultrasound alone vs. ultrasound + DS System: AUC 0.73 vs. 0.82, ORH shift +0.08 (USA +0.07, Spain +0.06), SEN +8%, SPE +14%, time −24%, r_p_ 0.62 → 0.88, κ 0.44 → 0.60; all results *p* < 0.001.	Significant improvement in AUC, sensitivity, specificity, interreader agreement, and efficiency through DS System assistance
**Ashton et al., 2024** [30]	retrospective, PACS workstation	347	649	USA	DS System vs. radiologist: AUC 0.70 vs. 0.70; SPE 63% vs. 43%; SEN 69% vs. 81%; combined (radiologist + DS System): AUC 0.76, SPE 53%, 17% fewer FNA recommendations	DS System alone comparable to radiologists (lower SEN, higher SPE); best performance in combination with radiologic assessment
**Carnabatu et al., 2024** [36]	retrospective, PACS workstation	27	28	USA	radiologist vs. DS System: AUC 0.71 vs. 0.86; κ 0.25; radiologist: SEN 100%, SPE 33%, PPV 33%, NPV 100%; DS System: SEN 86%, SPE 76%, PPV 55%, NPV 94%	DS System improved PPV with minimal loss in NPV; 50% of FNA recommendations changed
**Fernández Velasco et al., 2024** [31]	retrospective, PACS workstation	172	172	Spain	endocrinologist vs. DS System: AUC 0.78 vs. 0.82 (*p* < 0.001); SEN 82% vs. 87%; SPE 38% vs. 55%; NPV 95% vs. 96%; PPV 14% vs. 16%; ACC 43% vs. 49%; κ 0.74 → 0.98	DS System improved AUC, SEN, SPE, NPV, PPV, and interobserver agreement
**Fernández Velasco et al., 2025** [35]	retrospective, PACS workstation	75	75	Spain	DS System vs. endocrinologist: solid composition 80% vs. 36% (*p* < 0.01); hypoechoic: 52% vs. 16% (*p* < 0.01); punctate echogenic foci: 10.7% vs. 1.3% (*p* < 0.05); ACR TI-RADS: r_p_ = 0.34; κ: composition 0.23 (<0.05), echogenicity 0.11 (ns), margins 0.05 (ns), echogenic foci 0.18 (*p* < 0.05), ACR TI-RADS 0.03 (ns)	DS System without endocrinologist oversight did not significantly improve risk stratification; tendency toward risk overestimation

**Abbreviations:** ACC, (diagnostic) accuracy; ACR TI-RADS, American College of Radiology Thyroid Imaging Reporting and Data System; AI, artificial intelligence; DS, decision support; AUC, area under the curve; FNA, fine needle aspiration; κ, Cohen’s kappa; ns, not significant; NPV, negative predictive value; ORH, Obuchowski–Rockette–Hillis method; PPV, positive predictive value; r_p_, Pearson’s correlation coefficient; SEN, sensitivity; SPE, specificity.

## Data Availability

The original contributions presented in this study are included in the article. Further inquiries can be directed to the corresponding author.

## Abbreviations

The following abbreviations are used in this manuscript:

**κ**	Cohen’s kappa agreement
ACR	American College of Radiology
AFTN(s)	autonomously functioning thyroid nodules
AUC	Area Under the Curve
AI	artificial intelligence
CI	confidence interval
CE	Conformité Européenne
DEGUM	Deutsche Gesellschaft für Ultraschall in der Medizin (German Society for Ultrasound in Medicine)
DS	decision support
FDA	(U.S.) Food and Drug Administration
FNA	fine-needle aspiration
n.s.	not significant
PACS	Picture Archiving and Communication System
r_p_	Pearson’s correlation coefficient
r_s_	Spearman correlation coefficient
RSS	Risk Stratification System
TN(s)	thyroid nodule(s)
TI-RADS	Thyroid Imaging Reporting and Data System
TR	ACR TI-RADS category (TR1–TR5)
